# Characterization of alternative splicing events and prognostic signatures in gastric cancer

**DOI:** 10.1186/s12935-024-03348-8

**Published:** 2024-05-11

**Authors:** Nan Zhu, Yupeng Zhao, Wenjing Yan, Lan Wei, Qingqing Sang, Jianfang Li, Bingya Liu, Beiqin Yu

**Affiliations:** 1 Department of General Surgery, Shanghai Key Laboratory of Gastric Neoplasms, Shanghai Institute of Digestive Surgery, Ruijin Hospital, Shanghai Jiao Tong University School of Medicine, Shanghai, 200025 China; 2 Gastroenterological Surgery, The affiliated Wuxi No. 2, People’s Hospital of Nanjing Medical University, Wuxi, 200240 China

**Keywords:** Gastric cancer, Alternative splicing, Survival-associated AS events, Prognosis, STAT3

## Abstract

**Background:**

Accumulating evidences indicate that the specific alternative splicing (AS) events are linked to the occurrence and prognosis of gastric cancer (GC). Nevertheless, the impact of AS is still unclear and needed to further elucidation.

**Methods:**

The expression profile of GC and normal samples were downloaded from TCGA. AS events were achieved from SpliceSeq database. Cox regression together with LASSO analysis were employed to identify survival-associated AS events (SASEs) and calculate risk scores. PPI and pathway enrichment analysis were implemented to determine the function and pathways of these genes. Kaplan-Meier (K-M) analysis and Receiver Operating Characteristic Curves were used to evaluate the clinical significance of genes of SASEs. Q-PCR were applied to validate the hub genes on the survival prognosis in 47 GC samples. Drug sensitivity and immune cell infiltration analysis were conducted.

**Results:**

In total, 48 140 AS events in 10 610 genes from 361 GC and 31 normal samples were analyzed. Through univariate Cox regression, 855 SASEs in 763 genes were screened out. Further, these SASEs were analyzed by PPI and 17 hub genes were identified. Meanwhile, using Lasso and multivariate Cox regression analysis, 135 SASEs in 132 genes related to 7 AS forms were further screened and a GC prognostic model was constructed. K-M curves indicates that high-risk group has poorer prognosis. And the nomogram analysis on the basis of the multivariate Cox analysis was disclosed the interrelationships between 7 AS forms and clinical parameters in the model. Five key genes were then screened out by PPI analysis and Differential Expression Gene analysis based on TCGA and Combined-dataset, namely STAT3, RAD51B, SOCS2, POLE2 and TSR1. The expression levels of AS in STAT3, RAD51B, SOCS2, POLE2 and TSR1 were all significantly correlated with survival by qPCR verification. Nineteen drugs were sensitized to high-risk patients and eight immune cells showed significantly different infiltration between the STAD and normal groups.

**Conclusions:**

In this research, the prognostic model constructed by SASEs can be applied to predict the prognosis of GC patients and the selected key genes are expected to become new biomarkers and therapeutical targets for GC treatment.

**Supplementary Information:**

The online version contains supplementary material available at 10.1186/s12935-024-03348-8.

## Background

Globally, gastric cancer (GC) remains the fifth leading malignancy and the fourth most common cause of cancer death [1, 2]. The global burden of this malignancy is projected to increase by 62% by 2040, according to a study published in 2022 [3]. Although GC has become a highly prevalent malignancy tumor in clinical practice, its pathogenic mechanism has not been fully clarified. Despite the fact that the pathogenic mechanism of GC has not been fully clarified, it has become a highly prevalent malignant tumor in clinical practice. Besides surgical treatment, drugs are the main form for GC treatment, but their effectiveness is unsatisfactory. In advanced stages of disease, trastuzumab and some immune checkpoint inhibitors are more effective for patients with HER2-positive and PDL1-positive GC [4]. Patients without a specific target can only be treated with conventional chemotherapy, but drug resistance in tumors is a pressing issue in advanced GC [5].

Proteins are the major components of cells and tissues, playing a critical role in many important events. Diversity of protein species is the foundation of functional diversity. Alternative splicing (AS) not only enables an individual gene to generate functionally distinct protein isoforms to expand gene coding capacity, but also quantitatively regulates the level of protein expression, which makes AS an effective post-transcriptional regulatory mechanism [6].

**Fig. 1:**
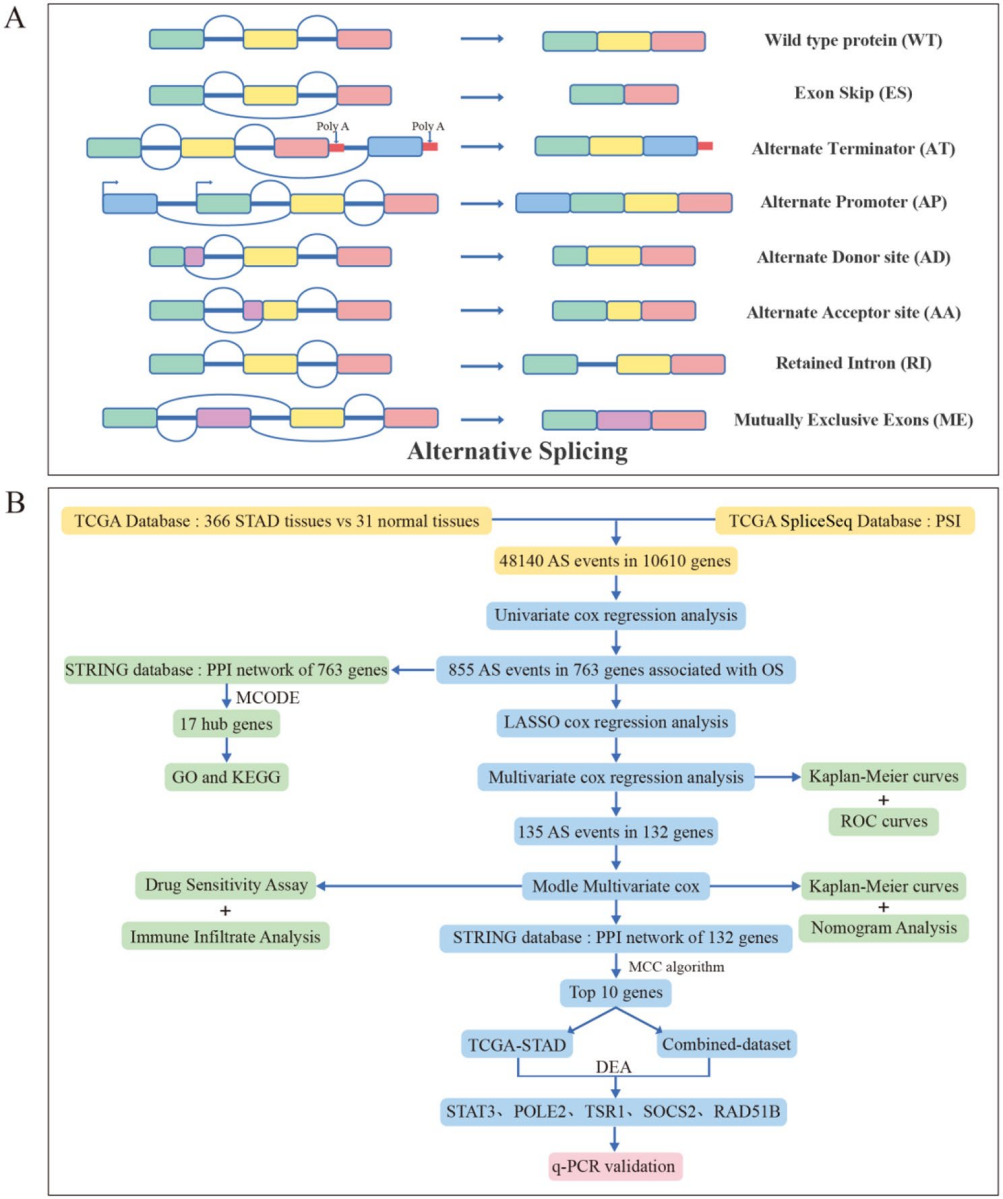
Patterns of AS and flowchart of this study. **A**: Schematic diagram of 7 AS forms including exon skipping (ES), alternate acceptor sites (AA), mutually exclusive exons (ME), alternate donor sites (AD), alternate terminator (AT), alternate promoter (AP) and retained intron (RI). **B**: The flowchart of this study. Download the expression and AS events data of GC and normal tissues from the TCGA database and SpliceSeq database. Univariate and multivariate Cox regression analysis together with LASSO analysis were employed to identify Survival-associated AS events(SASEs)and construct a prognostic model. STRING database were used to perform PPI analysis on genes of SASEs, GO and KEGG analysis were implemented to enrichment the function and pathways of these genes. K-M analysis and ROC curves were used to evaluate the clinical significance of genes of SASEs. Simultaneously conducting drug sensitivity analysis and immune cell infiltration analysis on the established prognostic model. Finally, q-PCR was used to validate the 5 selected hub genes and their AS variants

During physiological processes, AS alterations occur in over 95% of human multiexon genes [7]. As it is well known, there are 7 major types for AS, including Exon Skip (ES), Alternate Terminator (AT), Alternate Promoter (AP), Alternate Donor site (AD), Alternate Acceptor site (AA), Retained Intron (RI) and Mutually Exclusive Exons (ME) (Fig. 1A). The AS regulatory networks have a broad effect on the growth, development and differentiation of organisms [8]. The prevalence of abnormal AS events can bring about various diseases [9, 10], including cancer [11, 12]. A study showed that there were more than 30% AS events in tumor compared to normal samples [13]. Other reports showed the correlations between AS and the occurrence, development or metastasis of various tumors [11–15]. Therefore, understanding the AS of RNA in tumor will provide us an opportunity to further understand tumor biology, identify biomarkers for cancer diagnosis and treatment, and develop corresponding drugs to control tumor progression [16, 17]. Abnormal AS also can result in tumor drug resistance. In a study on melanoma, the authors discovered that a BRAF(V600E) splice variant that lacked the RAS binding domain could cause reduced sensitivity to vemurafenib [18]. As a subunit of U2 small nuclear ribonucleoprotein, SF3B promotes splice site recognition. This recognition can be inhibited by Pladienolide, FR901464 and some other compounds, resulting in the inhibition of in vitro splicing and the accumulation of pre-mRNA [19, 20]. Antisense oligonucleotide-based small molecule drugs are used to treatment by affecting the function of specific genes through exon skipping or intron retention, which can activate or inactivate their functions [21].

AS has important roles in GC development and multiple splice variants have been found associated with GC in recent years [22–25]. In the current study, we focus on AS events in TCGA-STAD and conducted bioinformatic analysis on GC and normal samples using TCGA and TCGA SpliceSeq databases. We identified hub survival-associated AS events (SASEs) and explored their potential mechanisms for influencing GC biological processes. Finally, clinical samples were used to validate the AS events of hub genes which can offer new insights for the diagnosis, targeted therapy and prognosis evaluation of GC patients. The workflow of this research illustrated in Fig. 1B.

## Methods

### Data extraction and pre-processing

The expression profile of the Stomach Adenocarcinoma dataset (STAD) which contains 448 GC samples and 36 normal samples were retrieved from The Cancer Genome Atlas (TCGA, https://portal.gdc.cancer.gov/) by using the R package TCGAbiolinks [26]. In total, 366 GC samples (STAD group) and 31 normal samples (normal group) with more than 30 days survival times were included in the present research (Supplementary Table 1).

The AS data of TCGA-STAD was downloaded from the TCGA SpliceSeq database (https://bioinformatics.mdanderson.org/TCGASpliceSeq) [27], and 7 types of AS events including ES, AT, AP, AD, AA, RI and ME were replaced with a Percentage Spliced In (PSI) from 0 to 1 for the subsequent analysis. This ratio reflects the proportional size of a particular AS event relative to the total splicing event of the genes. Using AA, one of the AS events, as an example, if 70% of the transcripts of a gene use AA as the primary splice acceptor site and the remaining 30% of the transcripts use other secondary acceptor sites, then the PSI value of the AA event in the sample is 0.7, reflecting that the vast majority of the transcripts prefer the primary splice acceptor site. If the PSI value of the sample is recorded as NA, it means that there is not enough data to estimate the frequency of this splicing event. In addition, to ensure that the sample data analyzed is complete and reliable, samples with a PSI value of NA exceeding 30% were excluded. Next, a special normalization procedure was used to convert the raw counts values to TPM and adjust for the effects of gene length and sequencing depth to ensure data from different samples were comparable. The limma package was then used to correct and quantify the background normalization, and the batch effect was processed in combination with the SVA package to ensure that the data could be used for the combined dataset for subsequent analysis. Finally, 392 AS samples were obtained, including 361 GC samples and 31 normal samples. GSE62254, GSE66222 and GSE15459 which respectively included 300, 100 and 100 GC samples were downloaded from GEO using GEOquery package [28]. As all of these samples are Homo sapiens and the sample size of each dataset is sufficient, they were chosen as external validation datasets. The same Affymetrix platform were used to analysis the three datasets to ensure data consistency. The datasets GSE66222, GSE62254, and GSE15459 were normalize and merge by Limma package [29]. R package SVA (version 4.1.3) was used for de batch processing to obtain the final merged dataset which named as Combined-dataset.

### Establishment of GC prognostic model for AS related events

The correlation between the PSI value and overall survival (OS) for each AS event in GC was calculated using univariate Cox regression analysis and only Hazard Ratio (HR) > 1 and AS events with a *P* value < 0.05 were retained. In order to construct a reliable prognostic model, only the most significant 50 AS events in the univariate Cox regression analysis were included, and then filter these top 50 most significant AS events using R’s glmnet package [30]. Further, LASSO regression analysis was performed on 7 different AS forms respectively [31]. In Lasso regression, tuning parameter (λ) is an adjustment parameter used to control the degree of feature selection. During the model establishment process, as λ increases, the selected feature parameters decrease, and the absolute value of the coefficient increases accordingly. We obtained two models: the simplest model and the best model, after simulation and selection of the number of features. We then selected the corresponding variables in the simplest model. Multivariate Cox regression analysis was used for further screening, and based on the Cox regression coefficient, the risk score (RS) formula is calculated as follows:$$\begin{gathered} RS = \left( {PS{I_{AS1}}{\text{*}}coe{f_{AS1}}} \right) + \left( {PS{I_{AS2}}coe{f_{AS2}}} \right) +  \hfill \\\ldots  + \left( {PS{I_{ASn}}{\text{*}}coe{f_{ASn}}} \right) \hfill \\ \end{gathered}$$. Kaplan-Meier (K-M) analysis and logarithmic rank test were performed using R packet survival, with samples divided into high and low groups based on the median RS [32]. In addition, time-dependent Receiver operating characteristic (ROC) curves were used to evaluate patients’ survival through calculating the area under the curve (AUC) value by time ROC R package [33]. The AUC value has lower accuracy when the area under it is between 0.5 and 0.7, a certain accuracy when it is between 0.7 and 0.9, and a higher accuracy when it is above 0.9. Multivariate Cox risk regression analysis with seven prognostic models of AS events, along with age, gender, tumor clinical stage and other relevant information was conducted to assess whether the final prognostic model can serve as independent prognostic factors for GC.

### PPI network analysis and hub genes identification

Protein-Protein Interaction (PPI) network was constructed for selected genes using Search Tool for the Retrieval of Interaction Gene/Proteins (STRING) online tool, and the least required interaction score in the STRING database was selected as low confidence (0.40) [34]. The PPI network analysis was used on two occasions in the study for the screening of prognostic genes. The first PPI network analysis which targeted all genes of SASEs screened from univariate Cox regression analysis used the Molecular Complex Detection (MCODE) plugin in Cytoscape (degree cutoff = 2, k-core = 2 and node score cutoff = 0.2) [35]. The second PPI network used MCC algorithm to screen hub genes from 135 SASEs in 132 genes in the prognostic model [36].

### KEGG and GO pathway analysis

The Kyoto Encyclopedia of Genes and Genomes (KEGG) is a well-known database of genomes, biological pathways, diseases and drugs information [37]. The Gene Ontology (GO) analysis is widely used to perform large-scale functional enrichment of biological processes (BP), molecular functions (MF) and cellular components (CC) [38]. ClusterProfiler [39] R package was used to perform KEGG pathway enrichment analysis and GO annotation analysis on hub genes, and use p.adj < 0.05 and qvalue < 0.25 as conditions to screen and display the first three results of BP, CC and MF in GO and KEGG pathways.

### Drug sensitivity analysis

The alterations in the cancer genome can be useful biomarkers for drug response and have a significant impact on the clinical response to treatment. The genomics of drug sensitivity in cancer (GDSC) database (https://www.cancerRxgene.org) is a public resource that provides molecular markers of drug response and information on drug sensitivity in cancer cells that can be used to search for sensitive biomarkers of the genome and cancer drug response data [40]. The pRRophytic algorithm was used to predict the sensitivity of patients with GC to commonly used anticancer drugs or to small molecule compounds in risk score high and low groups of 7 AS events combined with clinical parameters by calculating the IC50 value on the basis of the expression of GC samples, and the results are presented through group comparison graphs.

The Cancer Therapeutics Response Portal (CTRP) database (https://portals.broadinstitute.org/ctrp/) contains omics data, including mutations, gene expression and copy number variation, etc., from 860 cancer cell lines. Additionally, it includes drug sensitivity data from 481 small molecules drugs. This data can be used to correlate the genetics, lineage and other cellular characteristics of cell lines with small molecule drug sensitivities in order to accelerate the discovery of patient-matched cancer treatment molecules (drugs). The GSCA (Gene Set Cancer Analysis) database (http://bioinfo.life.hust.edu.cn/GSCA) integrates more than 10 000 multidimensional genomic data from TCGA for 33 cancer types and more than 750 small molecule drugs from GDSC and CTRP [41]. The hub genes (C18orf21, FYN, NFATC1, POLD4, POLE2, RAD51B, SOCS2, STAT3, TSC2, TSR1) were entered into the GSCA to obtain the correlation with drug susceptibility in CTRP and GDSC. The results were displayed by bubble charts.

### Immune cells infiltration analysis

The RNA seq dataset of STAD and corresponding normal samples were uploaded to CIBERSORT, combined with the LM22 feature gene, filtered out samples with *P* < 0.05, and obtained the immune cells infiltration. The gastric adenocarcinoma (STAD) RNA sequencing dataset and corresponding normal samples were uploaded to CIBERSORT and the LM22 feature gene was used to filter out samples with a p-value less than 0.05, resulting in the identification of immune cell infiltration. The distribution of 22 types of immune cells infiltration, including Naive B cells (NBC), memory B cells (Bmem), Plasma cells, CD8^+^ T cells, CD4^+^ Naive T Cells, resting CD4^+^ memory T cells, activated CD4^+^ memory T cells, T follicular helper cells (Tfh), regulatory T cells (Tregs), gamma delta T cells, resting NK cells, activated NK cells, Monocytes, M0 Macrophages, M1 Macrophages, M2 Macrophages, resting Dendritic cells, activated Dendritic cells, resting Mast cells, activated Mast cells, Eosinophils and Neutrophils, in each sample was demonstrated by plotting a bar chart using the R ggplot2 package. A correlation heatmap was drew to reflect the correlation among immune cells. Subsequently, by comparing the scores, immune cells were obtained from different infiltration levels between STAD and normal groups.

### Q-PCR and PCR analysis for AS events

Forty-seven fresh GC specimens from Ruijin Hospital, Shanghai Jiao Tong University School of Medicine were collected for validation. All these patients provided signed informed consent and they received no adjuvant treatment prior to the surgery. Primers were designed (primer sequences listed in Supplementary Table 2), and q-PCR was performed on the basis of the mRNA of 5 hub genes and their corresponding AS events. First, the above 47 fresh GC specimens were all extracted with TRIzol reagent (Invitrogen, Waltham, USA) for total RNA, and then cDNA was synthesized using the reverse transcription kit HiScript II Q Select RT SuperMix (Vazyme, Nanjing, China) according to the manufacturer’s instructions. Q-PCR was performed using ChamQ Universal SYBR qPCR Master Mix (Vazyme) kit for 40 cycles (95 °C for 3 s and 60 °C for 30 s). GAPDH was used as an internal control and the relative expression of mRNA level was quantified using the 2^-ΔCT^ method.

### Statistical analysis

The study applied K-M analysis to compare the ability of prognostic signatures to predict the outcome of GC patients, and ROC curves were drawn to demonstrate the efficiency of the prognostic signatures. All data statistical analyses are conducted with R (https://www.r-project.org/Version 4.2.2). The independent Student t-test was used to estimate statistical significance for normally distributed variables, while the Wilcoxon rank sum test was used to analyze differences between non-normally distributed variables when comparing two sets of continuous variables. All statistical tests were performed by 2-sided test. *P* values less than 0.05 were considered statistically significant.

## Results

### Landscape of AS events and SASEs in GC

**Fig. 2:**
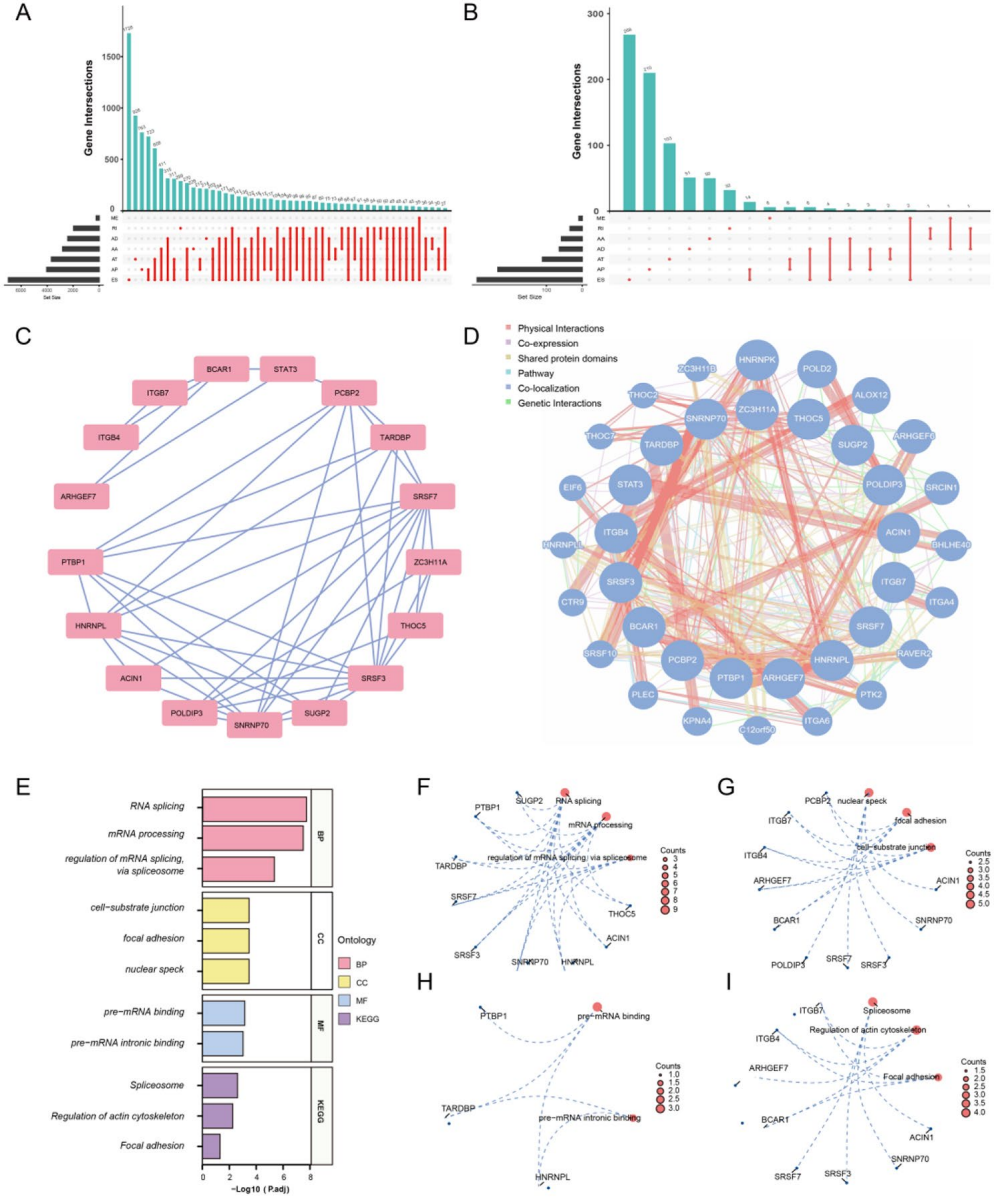
Overview of AS events in GC and GO and KEGG pathway. **A**: Upset plots of 7 AS events in STAD. **B**: Upset plots of OS related 7 AS events in STAD. **C**: Hub genes obtained based on MCODE plugin. **D**: Functionally similar gene interaction networks in hub genes. **E**: GO and KEGG pathways analysis of hub genes. **F-I**: The circular network diagrams of genes and pathways in the GO and KEGG analysis: BP pathway (**F**), CC pathway (**G**), MF pathway (**H**) and KEGG pathway (**I**), which blue dots representing specific genes and red blocks representing specific pathways

We obtained a total of 48 140 AS events in 10 610 genes from the TCGA Splice-Seq database, suggesting that AS is a common biological process in GC. A total of 48 140 AS events were obtained from the TCGA Splice-Seq database, occurring in 10 610 genes, indicating that AS is a prevalent biological process in GC. In detail, 19 121 ES events in 6972genes, 10 004 AP events in 4025genes, 8390 AT events in 3666 genes, 4006 AA events in 2799 genes, 3450 AD events in 2401 genes, 2944 RI events in 1956 genes and 226 ME events in 219 genes were observed in preliminary analysis, which showing that ES was the prevalent type and a single gene may have more than one type of mRNA splicing event (Fig. 2A).

To assess the prognostic importance of AS events in GC patients, univariate Cox regression analysis was performed to evaluate the correlation between PSI scores of each AS event and OS in GC, and 855 SASEs events in 763 genes were found to be significantly associated with the OS of GC patients, including 335 ES events in 294 genes, 238 AP events in 236 genes, 112 AT events in 111 genes, 67 AD events in 64 genes, 59 AA events in 58 genes, 35 RI events in 34 genes and 9 ME events in 9 genes (Fig. 2B).

### Hub genes of SASEs in GC and the analysis of GO and KEGG pathway

On the one hand, using the STRING database, we constructed a PPI network of 763 genes. From this network, 17 genes (BCAR1, STAT3, THOC5, ACIN1, HNRNPL, SNRNP70, SRSF3, SRSF7, ARHGEF7, TARDBP, PTBP1, ZC3H11A, ITGB4, ITGB7, PCBP2, SUGP2, POLDIP3) were identified as hub genes using the MCODE plug-in in Cytoscape (Fig. 2C). Functionally similar gene interaction networks in hub genes were predicted by GeneMANIA (Fig. 2D). Meanwhile, to clarify the function of genes with SASEs, we performed KEGG pathway and GO enrichment analyses. (Fig. 2E). GO analysis suggested that hub genes are involved in biological processes such as RNA splicing, mRNA processing and regulating mRNA splicing via the spliceosome (Fig. 2F), cell components such as nuclear specks, focal adhesions and cell substrate junctions (Fig. 2G), and molecular functions such as pre-mRNA binding, pre-mRNA intronic binding, and so on (Fig. 2H). The KEGG analysis revealed that 17 genes were enriched in pathways associated with cancer, including Spliceosome, Regulation of actin cytoskeleton and Focal adhesion (Fig. 2I, Supplementary Table 3).

### Constructing a GC prognostic model using SASEs and clinical parameters

**Fig. 3:**
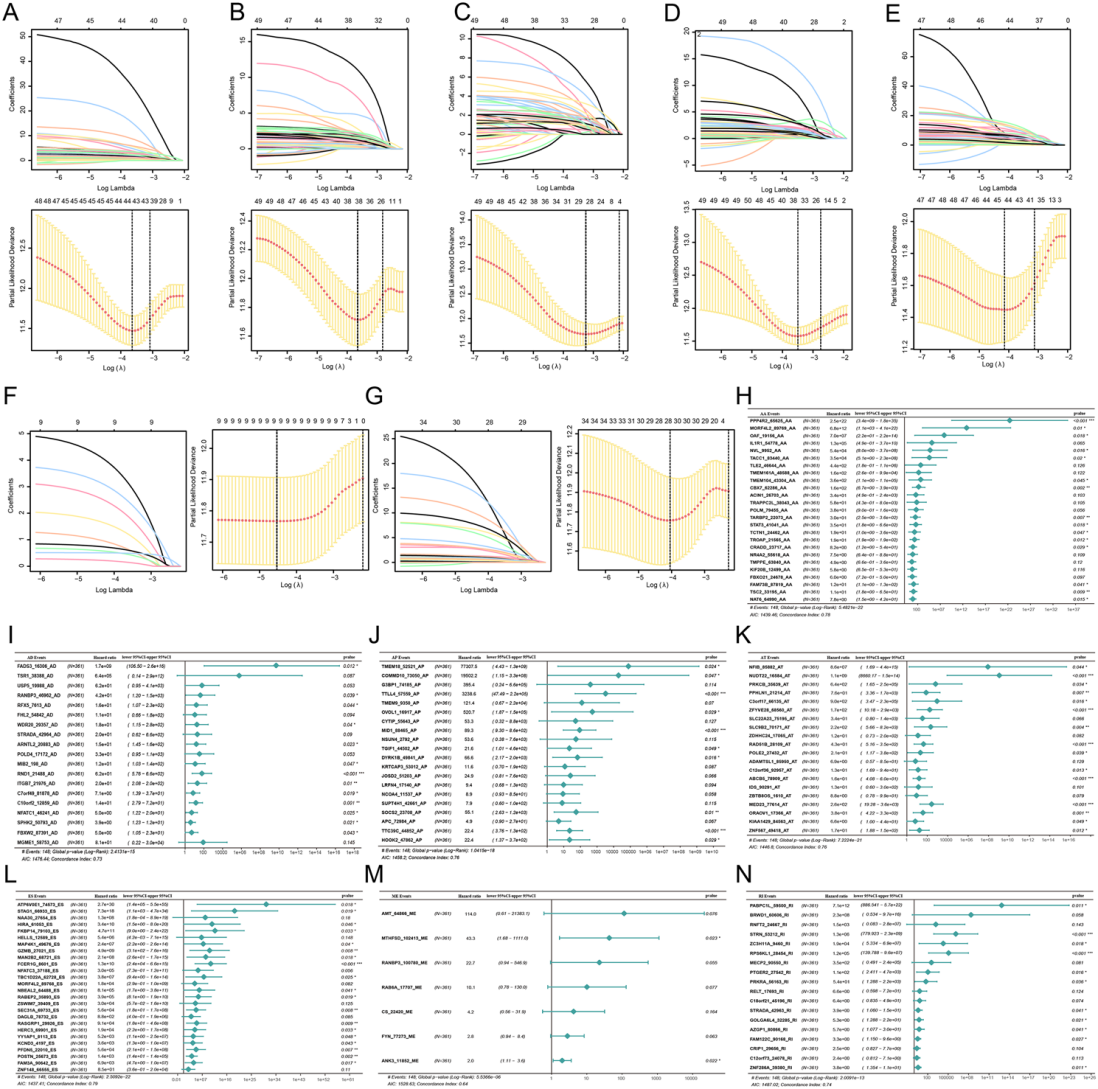
LASSO regression analysis and Forest map. **A-G**: LASSO regression analysis of seven types of AS events: AA (A), AD (B), AP (C), AT (D), ES (E), ME (F) and RI (G). **H-N**: Seven forest maps in multivariate Cox risk regression analysis in GC, namely AA (H), AD (I), AP (J), AT (K), ES (L), ME (M) and RI (N). The forest map includes AS events, sample size, risk ratio, 95% confidence interval and *P*-value (*: *P* 0.05; **: *P* 0.01; ***: *P* 0.001)

On the other hand, to develop the disease characteristics of GC and assess its diagnostic ability for the disease, LASSO Co1 analysis was carried out to establish an efficient prediction model using 855 SASEs events in 763 genes based on4ES, AP, AT, AA, AD, RI and ME events. After the simulation and the selection of the number of features, we selected the corresponding variables in the simplest model which corresponds to 43 AA events (Fig. 3A), 38 AD events (Fig. 3B), 28 AP events (Fig. 3C), 38 AT events (Fig. 3D), 44 ES events (Fig. 3E), 9 ME events (Fig. 3F) and 28 RI events (Fig. 3G) for subsequent analysis. The AS events linked to prognosis underwent additional screening through multivariate Cox regression analysis. Eventually, we obtained 135 SASEs events in 132 genes including 25 AA events (Fig. 3H), 19 AD events (Fig. 3I), 20 AP events (Fig. 3J), 20 AT events (Fig. 3K), 26 ES events (Fig. 3L), 7 ME events (Fig. 3M) and 18 RI events (Fig. 3N) to develop AS prognostic models (Supplementary Tables 4–5).

**Fig. 4:**
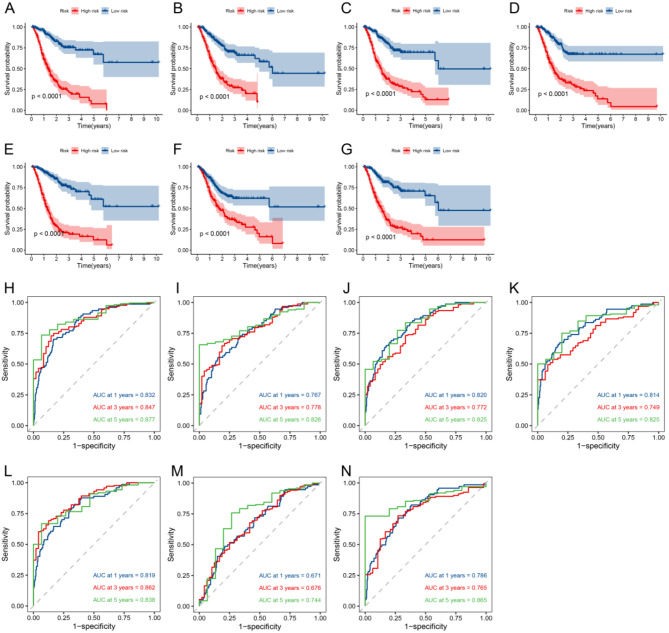
K-M curves and time dependent ROC curves of the GC prognostic model. **A-G**: In the GC prognostic model, the K-M analysis of AA (A), AD (B), AP (C), AT (D), ES (E), ME (F) and RI (G) shows that, red represents the high-risk group and blue represents the low-risk group. **H-N**: The time dependent ROC curves of AA (H), AD (I), AP (J), AT (K), ES (L), ME (M), and RI (N) in the GC prognostic model. AUC between 0.5 and 0.7, low accuracy; between 0.7 and 0.9, certain accuracy; above 0.9, high accuracy

**Fig. 5:**
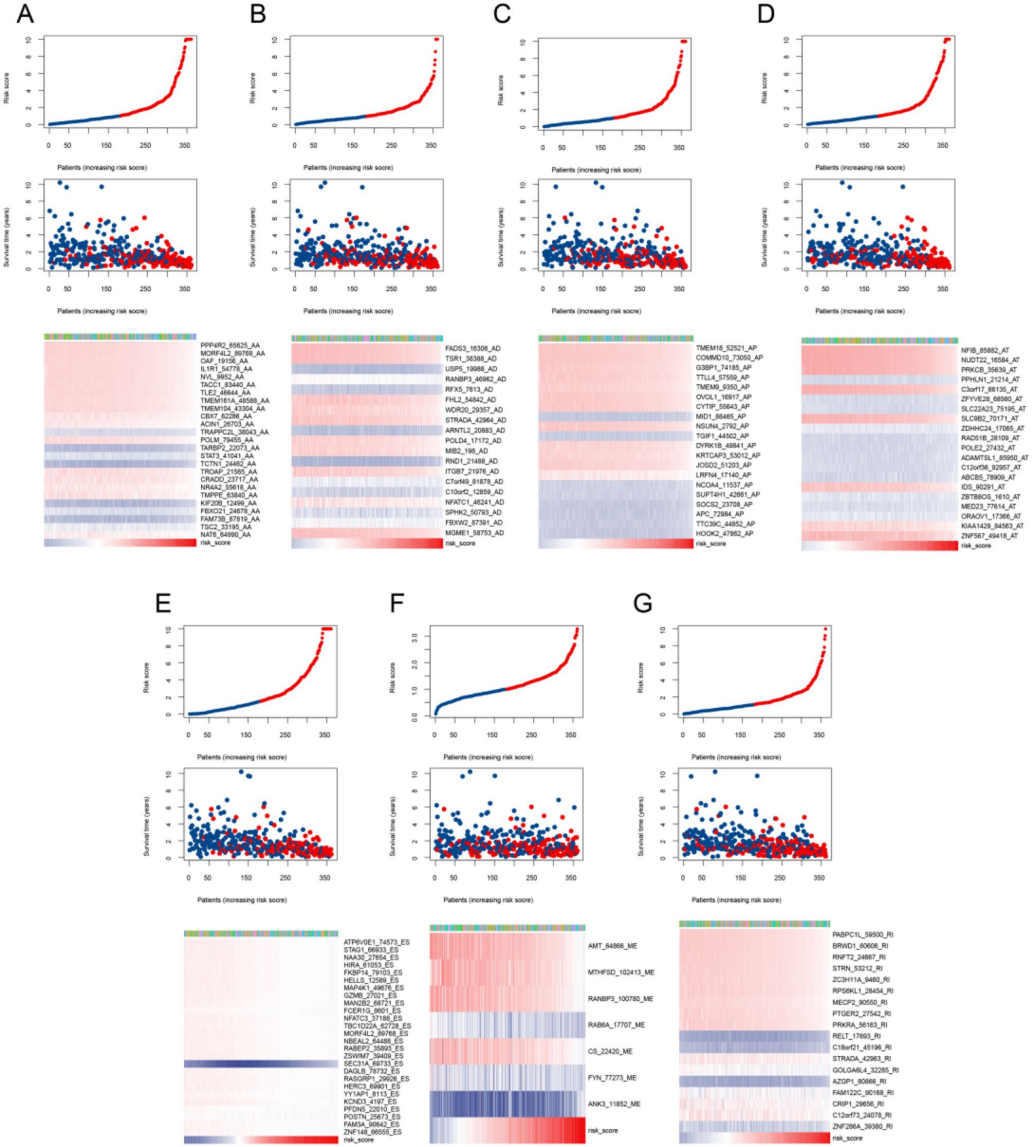
Triple plots of risk factors for the prognostic model in GC. **A-G**: Risk factor maps of AA (A), AD (B), AP (C), AT (D), ES (E), ME (F) and RI (G) in the GC prognostic model, including risk score (upper), survival status (middle) and PSI expression profile (lower)

After constructing the model, GC samples were then divided into two groups on the basis of their median score: the high-risk and low-risk groups. K-M survival analysis showed that each prognostic model for AS events in GC effectively discriminated survival curves between low-risk and high-risk groups (*P* < 0.05), with low-risk patients having longer survival (Fig. 4A-G). Subsequently, we plotted time dependent ROC curves for GC and calculated the AUC to test the accuracy of each model’s predictions over 1-, 3- and 5-year. It was found that the models for AA events (Fig. 4H), AD events (Fig. 4I), AP events (Fig. 4J), AT events (Fig. 4K), ES events (Fig. 4L) and RI events (Fig. 4N) had certain accuracy (AUC > 0.7) at 1-, 3- and 5- year, and the ME event prognostic model (Fig. 4M) has certain accuracy in the 5-year. The risk scores (RS) of seven prognostic model in GC showed that with the increase of RS, the proportion of dead patients also increases, while the proportion of surviving patients decreases (Fig. 5A-G).

**Fig. 6:**
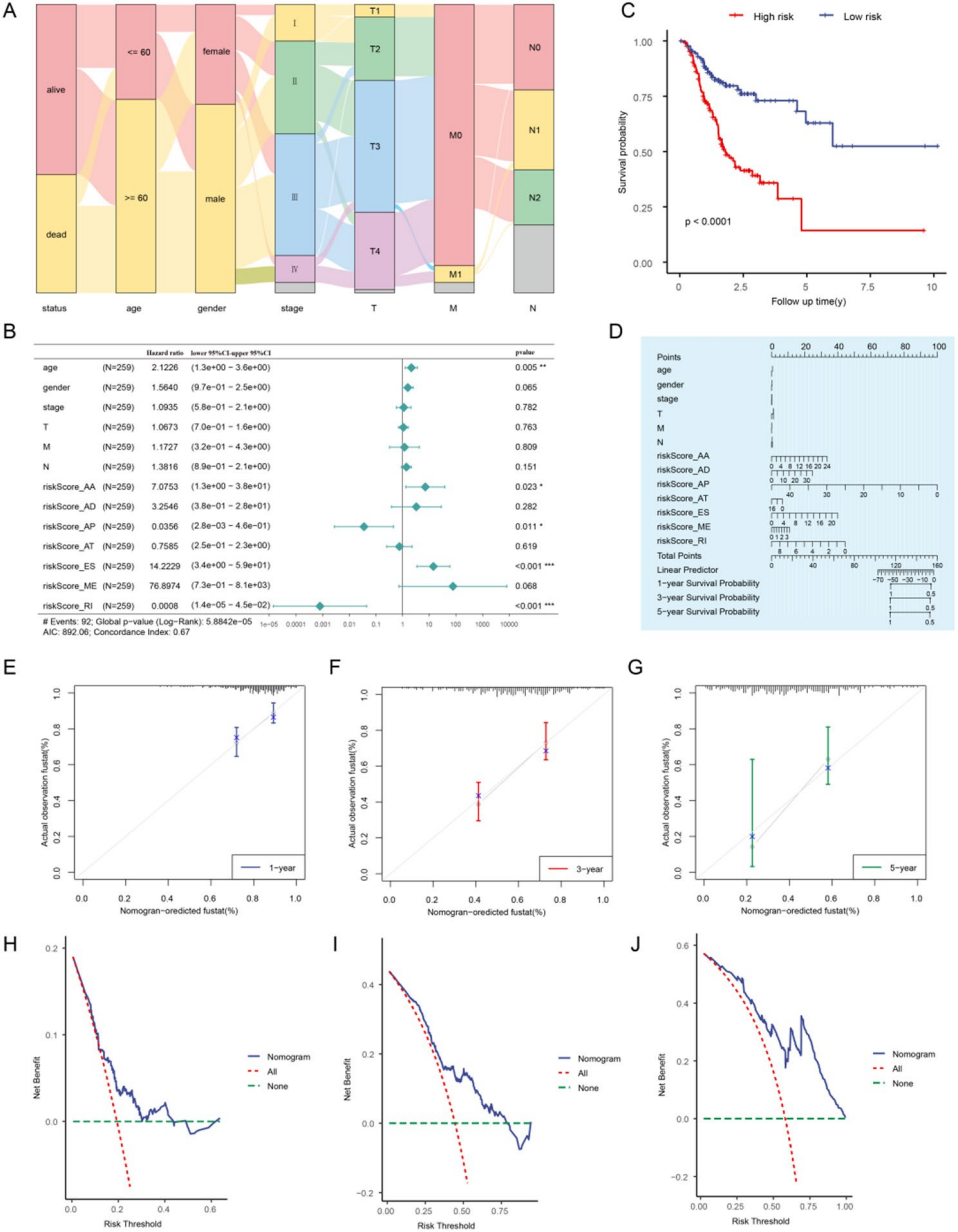
Prognostic analysis combined with clinical parameters in GC. **A**: Sankey plots of clinical variables in GC samples. **B**: Forest plots of multivariate Cox analysis. **C**: The K-M curve of prognostic model. **D**: Nomogram of prognostic analysis. **E-G**: Prognostic calibration curves of the model at 1-, 3- and 5-year. **H-J**: Decision Curve Analysis (DCA) plots of the model at 1-, 3- and 5-year, respectively. (*: *P* < 0.05; **: *P* < 0.01; ***: *P* < 0.001)

Determine if the final prognostic model can serve as an independent prognostic factor in the prognosis of GC, seven models for the prognosis of AS events were used together with clinical information such as age, gender and clinical stage in a multivariate Cox risk regression analysis. The clinical information was presented in a Sankey diagram (Fig. 6A). Prognostic models based on AA, AP, ES and RI were also found to be independent prognostic factors in multivariate Cox analysis in GC. (*P* < 0.05) (Fig. 6B, Supplementary Table 6), and the K-M curve was plotted on the basis of the high-risk and low-risk groups (Fig. 6C). Then, we conducted nomogram analysis on the gene and clinical information and a chart was plotted to determine its predictive ability (Fig. 6D). Furthermore, we performed a prognostic calibration analysis at 1-year (Fig. 6E), 3-year (Fig. 6F) and 5-year (Fig. 6G) based on the nomogram of the multivariate Cox regression model. The results showed that the blue line corresponding to 1-year is the closest to ideal gray scenario line, indicating that the forecasting performance of the model is better in the 1-year than in the 3-year and 5-year. In addition, the clinical utility of the constructed Cox regression prediction model at 1 (Fig. 6H), 3 (Fig. 6I) and 5 (Fig. 6J) year was evaluated using Decision Curve Analysis (DCA), and found the following: the blue line representing the prognostic model is significantly higher than the red line for all positive and the green line for all negative, and is more pronounced in the 5-year compared to the 1- and 3-year.

### PPI network analysis of genes in the prognostic model

**Fig. 7:**
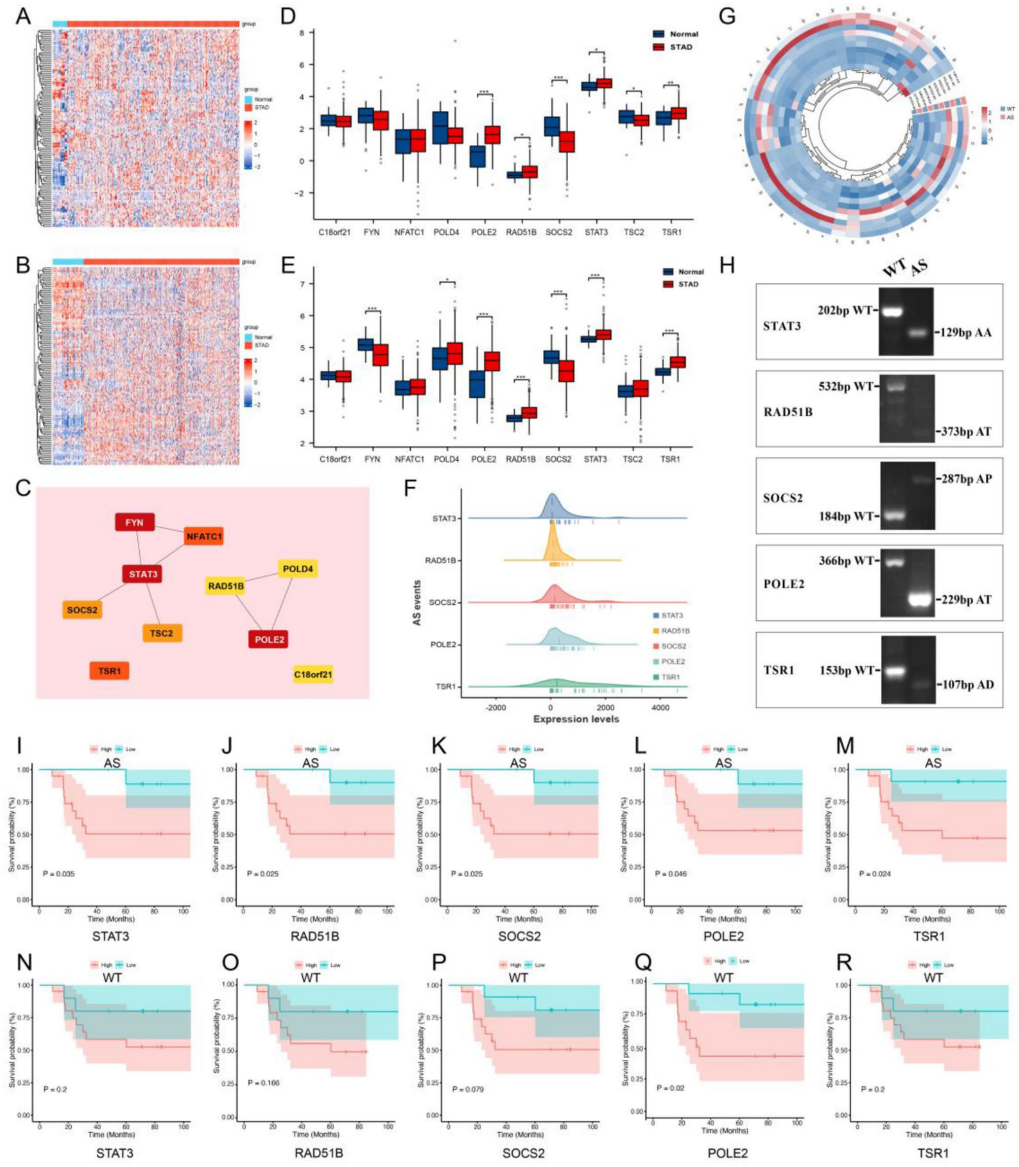
Q-PCR validation of hub genes of SASEs. **A**: Heatmaps of 132 prognostic related genes in GC. **B**: Heatmaps of 132 prognostic related genes in Combined-dataset. **C**: PPI network with 8 edges for 10 genes which was established using STRING and MCC algorithm among 132 genes. **D-E**: The comparison histogram of the differences between two groups of genes with TOP10 MCC score in STAD (D) and Combine-dataset (E). **F**: AS levels of 5 key genes in 47 clinical samples. **G**: Heatmaps of 5 hub genes in 47 clinical samples. **H**: PCR electrophoresis bands of 5 genes. **I-M**: K-M curves of genes STAT3-AA-41,041 (G), RAD51B-AT-28,109 (H), SOCS2-AP-23,708 (I), POLE2-AT-27,432 (J) and TSR1-AD-38,388 (K). **N-R**: K-M curves of genesSTAT3-WT (L), RAD51B-WT (M), SOCS2-WT (N), POLE2-WT (Q) and TSR1-WT (P)

First, to further screen key genes in GC prognostic models established above, a total of 132 corresponding genes were extracted. We plotted the expression heatmap of 132 prognosis-related genes in STAD and normal samples (Fig. 7A) and the heatmaps in the combined GEO datasets were also plotted (Fig. 7B). Subsequently, a PPI network among these 132 genes was established by using STRING. Next, the scores of the genes in the PPIs network were calculated using the MCC algorithm, and the genes in the network were ordered by gradient from red to yellow based on their scores. We selected the TOP10 genes (FYN, STAT3, POLE2, NFATC1, TSR1, TSC2, SOCS2, C18orf21, POLD4, RAD51B), and visualization of the network with Cytoscape software (Fig. 7C). Finally, we plotted the expression levels of ten genes in GC (Fig. 7D) and Combined dataset (Fig. 7E). And among the two datasets, there are 5 genes with consistent and statistically significant, namely STAT3, RAD51B, SOCS2, POLE2 and TSR1. According to the results of the prognostic model, the SASEs for these five genes are STAT3-AA-41,041, RAD51B-AT-28,109, SOCS2-AP-23,708, POLE2-AT-27,432 and TSR1-AD-38,388, respectively.

### Validation of key genes of SASEs in GC samples

In order to observe the impact of AS events corresponding to the 5 genes on the prognosis of GC, we collected 47 clinical samples of GC for validation. Subsequently, we designed corresponding primers (Supplementary Table 7) based on the WT and AS mRNA sequences and performed q-PCR validation on these selected hub genes of SASEs. The expressions of AS events for 5 genes in 47 GC samples were displayed in Fig. 7F, so as the heatmap showed in Fig. 7G. All of the amplified PCR electrophoresis bands of five genes were confirmed (Fig. 7H). On the basis of the median AS levels of these genes in GC samples, K-M curves showed that the higher AS levels of STAT3-AA-41,041 (Fig. 7I, *P* = 0.035), RAD51B-AT-28,109 (Fig. 7J, *P* = 0.025), SOCS2-AP-23,708 (Fig. 7K, *P* = 0.025), POLE2-AT-27,432 (Fig. 7L, *P* = 0.046) and TSR1-AD-38,388 (Fig. 7M, *P* = 0.024) were significantly correlated with short OS. Meanwhile, except for POLE2-WT (Fig. 7Q, *P* = 0.02), the K-M curves of STAT3-WT (Fig. 7N, *P* = 0.2), RAD51B-WT (Fig. 7O, *P* = 0.166), SOCS2-WT (Fig. 7P, *P* = 0.079) and TSR1-WT (Fig. 7R, *P* = 0.079) showed no statistically significant differences in 47 GC samples. The q-PCR results indicated that the AS variable in STAT3, RAD51B, SOCS2 and TSR1, may affect the prognosis of GC.

### Drug sensitivity analysis for 7 AS events in the prognostic model

**Fig. 8:**
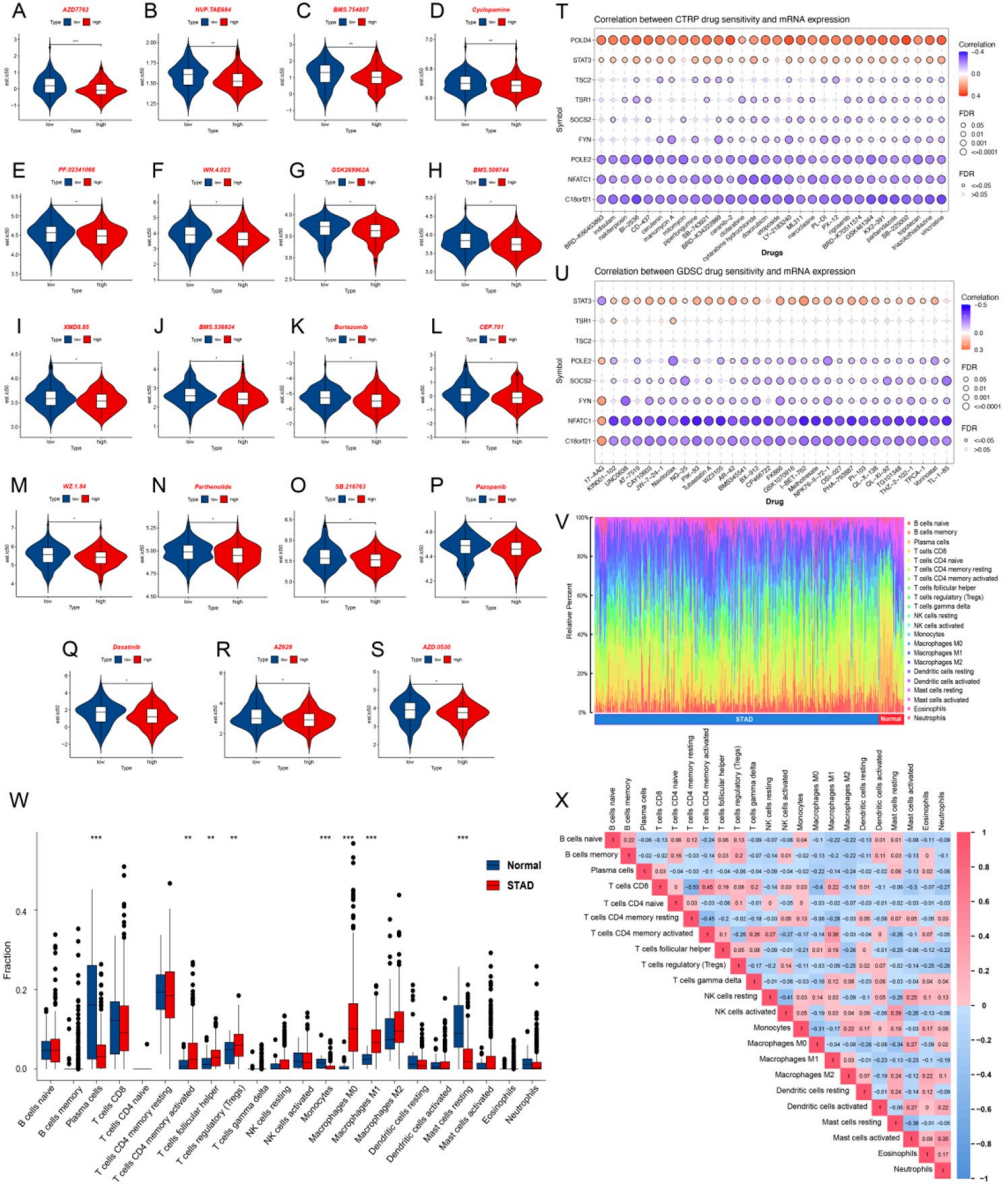
Analysis of drug sensitivity and Immune infiltration analysis. **A-S**: A chart of sensitivity analysis for high and low risk groups of the prognostic model including drug AZD7762, NVP.TAE684, BMS.754,807, Cyclopamine, PF.02341066, WH.4.023, GSK269962A, BMS.509,744, XMD8.85, BMS.536,924, Bortezomib, CEP.701, WZ.1.84, Parthenolide, SB.216,763, Pazopanib, Dasatinib, AZ628 and AZD.0530. **T-U**: The results of drug susceptibility analysis of gene C18orf21, FYN, NFATC1, POLD4, POLE2, RAD51B, SOCS2, STAT3, TSC2 and TSR1 were based on CTRP database (T) and GDSC database (U). **V**: Abundance map of immune infiltration in 22 immune cells in STAD and normal samples. **W**: Histogram of differences in each type of immune cells between STAD and normal samples. **X**: Correlation heatmap between immune cells

To be able to explore appropriate treatment strategies for patients with high-risk and low-risk groups with 7 types of AS events in the prognostic model score, drug sensitivity data from the GDSC database was used as a training set in predicting the sensitivity of clinical samples to common anticancer drugs in the GC dataset. We evaluated IC50 values among different anticancer drugs in the high-risk and low-risk groups of the prognostic model scores in GC, wherein 19 drugs (AZD7762, NVP.TAE684, BMS.754,807, Cyclopamine, PF.02341066, WH.4.023, GSK269962A, BMS.509,744, XMD8.85, BMS.536,924, Bortezomib, CEP.701, WZ.1.84, Parthenolide, SB.216,763, Pazopanib, Dasatinib, AZ628, AZD.0530) exhibited significantly lower IC50 (*P* < 0.05) in the high-risk group, indicating that those patients appeared to be more sensitive to the chemotherapy regimens containing these drugs which further highlighting the importance of individualized treatment for GC patients (Fig. 8A-S). The correlations between hub genes (C18orf21, FYN, NFATC1, POLD4, POLE2, RAD51B, SOCS2, STAT3, TSC2, TSR1) and drug susceptibility in CTRP and GDSC were shown in Fig. 8T-U.

### Immune cell infiltration analysis and its correlation

Using CIBERSORT on the expression data in the TCGA-STAD dataset, we analyzed 22 types of immune cell infiltration. By removal of all cells with a score of 0, we obtained an abundance map of immunological infiltration in 22 immune cells in STAD and normal samples (Fig. 8V). The immune cells were also examined for significant differences in infiltration scores between the STAD and normal groups (Fig. 8W). Moreover, significant differences in the infiltration of immune cells and immune function of Plasma cells, activated CD4^+^ memory T cells, Tfh, Tregs, Monocytes, M0 Macrophages, M1 Macrophages and resting Mast cells were represented between the two groups. Subsequently, the correlation among 22 types of immune cells was analyzed and visualized (Fig. 8X). All these findings suggested that immune cells between STAD and normal groups have different infiltration levels.

## Discussion

The phenomenon of AS was first discovered and proposed by Gilbert in his study of the adenovirus exon genes in 1978 [42]. Upon modification by AS, pre-mRNA can form numerous protein subtypes with different regulatory and functional properties. Abnormal AS events can promote the proliferation, metastasis, survival and drug resistance of cancer cells [43]. For example, CD44 expresses multiple subtypes through the AS process, and compared to wild-type, CD44 variant forms are associated with tumor development and considered as potential therapeutic targets for cancer treatment [44]. Nakka et al. demonstrated that SMAR1 negatively regulates the AS of CD44 through HDAC6 mediated deacetylation of RNA-binding protein Sam68 [45]. Lee et al. reported that epithelial and mesenchymal isoform switches of LRRFIP2 correlates with metastatic potential of GC cells by ESRP1 regulation [46].

Benefiting from the contribution of TCGA and TCGA SpliceSeq databases, 392 samples, including 361 GC and 31 normal samples were included in this research. We identified 855 SASEs in 763 genes using univariate Cox regression analysis, and 17 hub genes were identified by PPI network analysis. And regulation of mRNA splicing via spliceosome, RNA splicing and mRNA processing in biological processes; nuclear specks, focal adhesions, and cell substrate junctions in cell components; pre-mRNA binding, pre-mRNA intronic binding in molecular functions were presented in the GO analysis. Some underlying mechanisms, such as Spliceosome, Regulation of the actin cytoskeleton and Focal adhesion, were also identified by KEGG enrichment analysis.

For the 855 SASEs, we used LASSO Cox analysis and multivariate Cox regression analysis to further screen, and together with clinical pathological information, a prognostic model containing 135 SASEs in 132 genes were constructed. According to the K-M survival analysis results, it can be concluded that in diagnostic models of AS events, survival was lower in high-risk groups than in low-risk groups, and good diagnostic performance was observed at 5-year (AUC > 0.7). Subsequently, we established a PPI network for the corresponding 132 genes in prognostic models, and selected the top ten genes based on the MCC algorithm score. Further, 5 genes with consistent and statistically significant trends in both TCGA-STAD and Combined datasets were recognized as candidates, namely STAT3, RAD51B, SOCS2, POLE2 and TSR1.

STAT3 is a member of the STAT family and it is activated through transient phosphorylation of cytoplasmic monomers [47]. And it can act as an oncogene to induce tumor development through promoting immune escape mechanisms. Studies have shown that its splicing variant STAT3β over-expression can induce apoptosis and inhibit tumor growth [48, 49]. Members of the RAD51 family have been shown to have roles in homologous recombination and DNA repair. RAD51B, RAD51C, RAD51D, XRCC2, XRCC3 and SWSAP1 are paralogs of mammalian RAD51, and these proteins form distinct sub-complexes such as CX3 (XRCC3 and RAD51C), BCDX2 (XRCC2, RAD51B, RAD51C and RAD51D), and the Shu complex (SWS1 and SWSAP1) [50, 51]. Both RAD51 family members and their AS events can promote the occurrence of tumors [52, 53]. Robert A et al. found that isoform 1 is the functional isoform of RAD51D, while isoform 4 with a large deletion at N-terminal and isoform 6 with an alternate exon at N-terminus, do not functional in tumors [54]. Zhang et al. found that RAD51 undergoes AS by the regulation of LncRNA CACClnc and promotes chemotherapy resistance in colorectal cancer [55]. The suppressor of cytokine signaling (SOCS) family (SOCS1-SOCS7 and CIS) has been identified as negative inhibitors of cytokine signaling [56]. Chen et al. demonstrated that high expression of SOCS2 enhances the sensitivity of HCC radiotherapy by promoting the ubiquitination degradation of SLC7A11 and promoting ferroptosis [57]. However, the role of AS events among members of the SOCS family in tumors has not received further research attention. The TCGA-STAD and Combined datasets indicated that the SOCS2 is expressed at lower levels in GC compared to normal tissues. In our results, the expression of its AS subtype SOCS2-AP-23,708 is negatively correlated with survival prognosis. Therefore, we speculate that the imbalanced expression of SOCS2-WT and SOCS2-AP-23,708 may be one of the factors leading to the occurrence of GC and affecting its prognosis. But this hypothesis still needs further experimental verification in the future. DNA polymerase epsilon subunit 2 (POLE2) is a nuclear DNA polymerase subunit that is commonly associated with DNA repair and is aberrantly expressed in cancer., such as renal cell carcinoma [58], colorectal cancer [59], bladder cancer [60] and lung adenocarcinoma [61]. In addition, Jian et al. found that POLE2 activates GPX4 and inhibits ferroptosis in GC cells by increasing the activity of NRF2 [62]. TSR1 is a kind of ribosome maturation factor. Sun et al. reported the role of multiple rare deleterious variants of TSR1 in spontaneous coronary artery dissection (SCAD) [63]. However, the role of TSR1 and its AS forms in tumors is not yet clear, this article may provide some hints on its function in GC.

All of these candidates were verified by q-PCR in 47 GC tissues, and their expression levels of AS in STAT3, RAD51B, SOCS2, POLE2 and TSR1 were significantly correlated with survival time. Honestly, several limitations need to be considered. First, our analysis of SASEs in GC was retrospective, based on the data from TCGA. To validate this model, large multicenter cohorts are necessary. Secondly, this study has not yet identified the upstream splicing factors that regulate SASEs, nor has it clarified the specific mechanism of splicing variants in the occurrence and development of GC. Additionally, the roles of these genes in GC prognosis are not yet clear, so there would be a lot of work to improve the mechanisms of AS in GC.

## Conclusions

On the basis of the data mining of TCGA-STAD, this project developed a prognostic model using SASEs and clinicopathological characteristics to provide a convincing prediction of the long-term survival outcomes of patients with GC, which provided a new perspective on gene regulation in the progression of GC through a comprehensive analysis of SASEs and gene expression.

### Electronic supplementary material

Below is the link to the electronic supplementary material.

Supplementary Material 1

## Data Availability

No datasets were generated or analysed during the current study.

## Abbreviations

AS: Alternative Splicing
GC: Gastric Cancer
TCGA: The Cancer Genome Atlas
SASEs: Survival-associated AS events
PPI: Protein-Protein Interaction
ROC: Receiver Operating Characteristic
q-PCR: Quantitative Real-time Polymerase Chain Reaction
EBV: Epstein–Barr virus
AA: Alternate Acceptor site
AD: Alternate Donor site
AP: Alternate Promoter
AT: Alternate Terminator
ES: Exon Skip
ME: Mutually Exclusive Exons
RI: Retained Intron
STAD: Stomach Adenocarcinoma
PSI: Percentage Spliced In
OS: Overall Survival
HR: Hazard Ratio
RS: Risk Score
MCODE: Molecular Complex Detection
GO: Gene Ontology
BP: Biological Processes
MF: Molecular Functions
CC: Cellular Components
KEGG: Kyoto Encyclopedia of Genes and Genomes
GDSC: Genomics of Drug Sensitivity in Cancer
GSCA: Gene Set Cancer Analysis
CTRP: Cancer Therapeutics Response Portal
Tregs: T regulatory cells
