# Supplementary material for: Characterization of alternative splicing events and prognostic signatures in gastric cancer
Source: Cancer Cell Int. 2024 May 11;24:167. doi: 10.1186/s12935-024-03348-8 (PMC11088037; doi:10.1186/s12935-024-03348-8)
Supplement: Supplementary file 1 — Supplementary Material 1 [file 12935_2024_3348_MOESM1_ESM.docx]

**Supplementary Table 1. Participants' clinical features.**

| **Characteristics** | **Overall** |
| --- | --- |
| OS, n (%) |  |
| dead | 159 (40.1%) |
| alive | 238 (59.9%) |
| age, n (%) |  |
| <= 65 | 268 (67.5%) |
| > 65 | 129 (32.5%) |
| gender, n (%) |  |
| male | 259 (65.2%) |
| female | 138 (34.8%) |
| stage, n (%) |  |
| 3 | 160 (41.9%) |
| 1 | 52 (13.6%) |
| 4 | 37 (9.7%) |
| 2 | 133 (34.8%) |
| T, n (%) |  |
| 4 | 102 (26%) |
| 2 | 91 (23.2%) |
| 3 | 181 (46.1%) |
| 1 | 19 (4.8%) |
| M, n (%) |  |
| 0 | 359 (94%) |
| 1 | 23 (6%) |
| N, n (%) |  |
| 0 | 119 (38.5%) |
| 1 | 112 (36.2%) |
| 2 | 78 (25.2%) |

**Supplementary Table 2. Primers of q-PCR.**

| **Primer name** | **Forward sequence** | **Reverse sequence** |
| --- | --- | --- |
| STAT3-WT | CTGATTTTAGCAGGATGGCC | CTCCCAGGAGATTATGAAACAC |
| STAT-AA | CCCCTGATTTTAGGATGGCC | CAATCCAAGGGGCCAGAA |
| RAD51B-WT | GGCTCCCTTCACCTCATTTGTCT | GGTGCCTTAGTTCACCTCCTGTTT |
| RAD51B-AT | GCTCCCTTCACCTCATTTGTCTACAC | CCCCTATCTGGTATAGCAGAGAGTTC |
| TSR1-WT | AAGGCCAGGGGATCTGCA | ACAGCTAGTGTATAGGTCGGAAG |
| TSR1-AD | AGCAAGGCCAGGGGATCT | CAGCTAGTGCGGTGCTGT |
| SOCS2-WT | AACCGAGGCCAGTCACCAA | TTCCTTGAAGTCAGTGCGAATCC |
| SOCS2-AP | ATCGATGTGTCTTAGAGCCGGA | AAGGGATGGGGCTCTTTCTC |
| POLE2-WT | TACACAGGAAATTACTGTCTTCCG | GAATCTCAAAAGCCTTGAAGTTTGC |
| POLE2-AT | CCTAGCAGCAATTTGGCTATTCCTAATC | CTACATTCTTACAGGGTTTATGCAGAGGC |

**Supplementary Table 3. GO and KEGG pathway analyses.**

| **Ontology** | **ID** | **Description** | **GeneRatio** | **BgRatio** | **pvalue** | **p.adjust** |
| --- | --- | --- | --- | --- | --- | --- |
| BP | GO:0008380 | RNA splicing | 9/17 | 438/18800 | 3.84e-11 | 1.96e-08 |
| BP | GO:0006397 | mRNA processing | 9/17 | 500/18800 | 1x.25e-10 | 3.19e-08 |
| BP | GO:0048024 | regulation of mRNA splicing, via spliceosome | 5/17 | 103/18800 | 2.63e-08 | 4.48e-06 |
| BP | GO:0051028 | mRNA transport | 5/17 | 126/18800 | 7.24e-08 | 9.25e-06 |
| BP | GO:0050684 | regulation of mRNA processing | 5/17 | 139/18800 | 1.18e-07 | 9.99e-06 |
| CC | GO:0016607 | nuclear speck | 5/17 | 411/19594 | 1.99e-05 | 0.0003 |
| CC | GO:0005925 | focal adhesion | 5/17 | 419/19594 | 2.19e-05 | 0.0003 |
| CC | GO:0030055 | cell-substrate junction | 5/17 | 428/19594 | 2.42e-05 | 0.0003 |
| CC | GO:0008305 | integrin complex | 2/17 | 31/19594 | 0.0003 | 0.0034 |
| CC | GO:0098636 | protein complex involved in cell adhesion | 2/17 | 43/19594 | 0.0006 | 0.0053 |
| MF | GO:0036002 | pre-mRNA binding | 3/17 | 58/18410 | 1.96e-05 | 0.0007 |
| MF | GO:0097157 | pre-mRNA intronic binding | 2/17 | 12/18410 | 5.27e-05 | 0.0009 |
| KEGG | hsa03040 | Spliceosome | 4/12 | 147/8164 | 4.46e-05 | 0.0024 |
| KEGG | hsa04810 | Regulation of actin cytoskeleton | 4/12 | 218/8164 | 0.0002 | 0.0056 |
| KEGG | hsa04510 | Focal adhesion | 3/12 | 201/8164 | 0.0027 | 0.0494 |
| KEGG | hsa05412 | Arrhythmogenic right ventricular cardiomyopathy | 2/12 | 77/8164 | 0.0055 | 0.0576 |
| KEGG | hsa04512 | ECM-receptor interaction | 2/12 | 88/8164 | 0.0071 | 0.0576 |

GO: Gene Ontology; BP: biological process; CC: cellular component; MF: molecular function; KEGG: Kyoto Encyclopedia of Genes and Genomes.

**Supplementary Table 4. List of genes related to prognosis.**

| **GENE** | **GENE** | **GENE** | **GENE** | **GENE** | **GENE** |
| --- | --- | --- | --- | --- | --- |
| PPP4R2 | NFATC1 | MED23 | FAM73B | TTC39C | HERC3 |
| MORF4L2 | SPHK2 | ORAOV1 | TSC2 | HOOK2 | YY1AP1 |
| OAF | FBXW2 | KIAA1429 | NAT6 | NFIB | KCND3 |
| IL1R1 | MGME1 | ZNF567 | FADS3 | NUDT22 | PFDN5 |
| NVL | TMEM18 | ATP6V0E1 | TSR1 | PRKCB | POSTN |
| TACC1 | COMMD10 | STAG1 | USP5 | PPHLN1 | FAM3A |
| TLE2 | G3BP1 | NAA30 | RANBP3 | C3orf17 | ZNF148 |
| TMEM161A | TTLL4 | HIRA | RFX5 | ZFYVE28 | AMT |
| TMEM104 | TMEM9 | FKBP14 | FHL2 | SLC22A23 | MTHFSD |
| CBX7 | OVOL1 | HELLS | WDR20 | SLC9B2 | RAB6A |
| ACIN1 | CYTIP | MAP4K1 | STRADA | ZDHHC24 | CS |
| TRAPPC2L | MID1 | GZMB | ARNTL2 | RAD51B | FYN |
| POLM | NSUN4 | MAN2B2 | POLD4 | POLE2 | ANK3 |
| TARBP2 | TGIF1 | FCER1G | MIB2 | ADAMTSL1 | PABPC1L |
| STAT3 | DYRK1B | NFATC3 | RND1 | C12orf36 | BRWD1 |
| TCTN1 | KRTCAP3 | TBC1D22A | ITGB7 | ABCB5 | RNFT2 |
| TROAP | JOSD2 | NBEAL2 | C7orf49 | IDS | STRN |
| CRADD | LRFN4 | RABEP2 | C10orf2 | ZBTB8OS | ZC3H11A |
| NR4A2 | NCOA4 | ZSWIM7 | GOLGA6L4 | C12orf73 | RPS6KL1 |
| TMPPE | SUPT4H1 | SEC31A | AZGP1 | ZNF286A | MECP2 |
| KIF20B | SOCS2 | DAGLB | FAM122C | RELT | PTGER2 |
| FBXO21 | APC | RASGRP1 | CRIP1 | C18orf21 | PRKRA |

**Supplementary Table 5. 135 AS events in the prognostic model.**

| **AA** | **AD** | **AP** | **AT** |
| --- | --- | --- | --- |
| PPP4R2_65625_AA | FADS3_16306_AD | TMEM18_52521_AP | NFIB_85882_AT |
| MORF4L2_89769_AA | TSR1_38388_AD | COMMD10_73050_AP | NUDT22_16584_AT |
| OAF_19156_AA | USP5_19988_AD | G3BP1_74185_AP | PRKCB_35639_AT |
| IL1R1_54778_AA | RANBP3_46962_AD | TTLL4_57559_AP | PPHLN1_21214_AT |
| NVL_9952_AA | RFX5_7613_AD | TMEM9_9350_AP | C3orf17_66135_AT |
| TACC1_83440_AA | FHL2_54842_AD | OVOL1_16917_AP | ZFYVE28_68560_AT |
| TLE2_46644_AA | WDR20_29357_AD | CYTIP_55643_AP | SLC22A23_75195_AT |
| TMEM161A_48588_AA | STRADA_42964_AD | MID1_88465_AP | SLC9B2_70171_AT |
| TMEM104_43304_AA | ARNTL2_20883_AD | NSUN4_2792_AP | ZDHHC24_17065_AT |
| CBX7_62286_AA | POLD4_17172_AD | TGIF1_44502_AP | RAD51B_28109_AT |
| ACIN1_26703_AA | MIB2_198_AD | DYRK1B_49841_AP | POLE2_27432_AT |
| TRAPPC2L_38043_AA | RND1_21488_AD | KRTCAP3_53012_AP | ADAMTSL1_85950_AT |
| POLM_79455_AA | ITGB7_21976_AD | JOSD2_51203_AP | C12orf36_92957_AT |
| TARBP2_22073_AA | C7orf49_81878_AD | LRFN4_17140_AP | ABCB5_78909_AT |
| STAT3_41041_AA | C10orf2_12859_AD | NCOA4_11537_AP | IDS_90291_AT |
| TCTN1_24462_AA | NFATC1_46241_AD | SUPT4H1_42661_AP | ZBTB8OS_1610_AT |
| TROAP_21565_AA | SPHK2_50793_AD | SOCS2_23708_AP | MED23_77614_AT |
| CRADD_23717_AA | FBXW2_87391_AD | APC_72984_AP | ORAOV1_17366_AT |
| NR4A2_55618_AA | MGME1_58753_AD | TTC39C_44852_AP | KIAA1429_84563_AT |
| TMPPE_63840_AA |  | HOOK2_47862_AP | ZNF567_49418_AT |
| KIF20B_12499_AA |  |  |  |
| FBXO21_24678_AA |  |  |  |
| FAM73B_87819_AA |  |  |  |
| TSC2_33195_AA |  |  |  |
| NAT6_64990_AA |  |  |  |
| **ES** | **ME** | **RI** |  |
| ATP6V0E1_74573_ES | AMT_64866_ME | PABPC1L_59500_RI |  |
| STAG1_66933_ES | MTHFSD_102413_ME | BRWD1_60606_RI |  |
| NAA30_27654_ES | RANBP3_100780_ME | RNFT2_24667_RI |  |
| HIRA_61053_ES | RAB6A_17707_ME | STRN_53212_RI |  |
| FKBP14_79103_ES | CS_22420_ME | ZC3H11A_9460_RI |  |
| HELLS_12589_ES | FYN_77273_ME | RPS6KL1_28454_RI |  |
| MAP4K1_49676_ES | ANK3_11852_ME | MECP2_90550_RI |  |
| GZMB_27021_ES |  | PTGER2_27542_RI |  |
| MAN2B2_68721_ES |  | PRKRA_56163_RI |  |
| FCER1G_8601_ES |  | RELT_17693_RI |  |
| NFATC3_37188_ES |  | C18orf21_45196_RI |  |
| TBC1D22A_62728_ES |  | STRADA_42963_RI |  |
| MORF4L2_89768_ES |  | GOLGA6L4_32285_RI |  |
| NBEAL2_64488_ES |  | AZGP1_80866_RI |  |
| RABEP2_35893_ES |  | FAM122C_90168_RI |  |
| ZSWIM7_39409_ES |  | CRIP1_29656_RI |  |
| SEC31A_69733_ES |  | C12orf73_24078_RI |  |
| DAGLB_78732_ES |  | ZNF286A_39380_RI |  |
| RASGRP1_29926_ES |  |  |  |
| HERC3_69901_ES |  |  |  |
| YY1AP1_8113_ES |  |  |  |
| KCND3_4197_ES |  |  |  |
| PFDN5_22010_ES |  |  |  |
| POSTN_25673_ES |  |  |  |
| FAM3A_90642_ES |  |  |  |
| ZNF148_66555_ES |  |  |  |

**Supplementary Table 6. Multivariate Cox analysis combined with clinical parameters.**

| **id** | **HR** | **HR.95L** | **HR.95H** | **pvalue** |
| --- | --- | --- | --- | --- |
| age | 2.122573 | 1.261758 | 3.570667 | 0.004567 |
| gender | 1.56396 | 0.972203 | 2.515906 | 0.06522 |
| stage | 1.093475 | 0.580742 | 2.058896 | 0.781955 |
| T | 1.067306 | 0.699302 | 1.628971 | 0.76269 |
| M | 1.1727 | 0.323024 | 4.257341 | 0.808648 |
| N | 1.381568 | 0.888854 | 2.147406 | 0.150898 |
| riskScore_AA | 7.075342 | 1.3109 | 38.18785 | 0.022925 |
| riskScore_AD | 3.254556 | 0.379308 | 27.9249 | 0.281918 |
| riskScore_AP | 0.035604 | 0.002756 | 0.459962 | 0.010623 |
| riskScore_AT | 0.758512 | 0.254957 | 2.256617 | 0.619276 |
| riskScore_ES | 14.22294 | 3.443569 | 58.74486 | 0.000244 |
| riskScore_ME | 76.89736 | 0.725985 | 8145.082 | 0.067947 |
| riskScore_RI | 0.000796 | 1.41E-05 | 0.044818 | 0.000521 |

HR: Hazard Ratio; AA: Alternate Acceptor site; AD: Alternate Donor site; AP: Alternate Promoter; AT: Alternate Terminator; ES: Exon Skip; ME: Mutually Exclusive Exons; RI: Retained Intron.

**Supplementary Table 7. The sequences of primers used for q-PCR.**

| **Gene** | **Primer** |
| --- | --- |
| STAT3-WT-F | CTGATTTTAGCAGGATGGCC |
| STAT3-WT-R | CTCCCAGGAGATTATGAAACAC |
| STAT3-AA-41041-F | CCCCTGATTTTAGGATGGCC |
| STAT3-AA-41041-R | CAATCCAAGGGGCCAGAA |
| RAD51B-WT-F | GGCTCCCTTCACCTCATTTGTCT |
| RAD51B-WT-R | GGTGCCTTAGTTCACCTCCTGTTT |
| RAD51B-AT-28109-F | GCTCCCTTCACCTCATTTGTCTACAC |
| RAD51B-AT-28109-R | CCCCTATCTGGTATAGCAGAGAGTTC |
| SOCS2-WT-F | AACCGAGGCCAGTCACCAA |
| SOCS2-WT-R | TTCCTTGAAGTCAGTGCGAATCC |
| SOCS2-AP-23708-F | ATCGATGTGTCTTAGAGCCGGA |
| SOCS2-AP-23708-R | AAGGGATGGGGCTCTTTCTC |
| POLE2-WT-F | TACACAGGAAATTACTGTCTTCCG |
| POLE2-WT-R | GAATCTCAAAAGCCTTGAAGTTTGC |
| POLE2-AT-27432-F | CCTAGCAGCAATTTGGCTATTCCTAATC |
| POLE2-AT-27432-R | CTACATTCTTACAGGGTTTATGCAGAGGC |
| TSR-WT-F | AAGGCCAGGGGATCTGCA |
| TSR-WT-R | ACAGCTAGTGTATAGGTCGGAAG |
| TSR1-AD-38388-F | AGCAAGGCCAGGGGATCT |
| TSR1-AD-38388-R | CAGCTAGTGCGGTGCTGT |
